# The three pathways of advanced glycation end product synthesis: Hodge, Namiki, and Wolff — a clinically oriented mechanistic synthesis with network-based implications

**DOI:** 10.3389/fendo.2026.1856036

**Published:** 2026-06-03

**Authors:** Enrique C. Fernandez

**Affiliations:** Kendall Regional Medical Center, Miami, FL, United States

**Keywords:** advanced glycation end products, age synthesis, clinical biomarkers, glyoxalase, Hodge pathway, mechanistic review, methylglyoxal, Namiki pathway

## Abstract

**Background:**

Advanced Glycation End Products (AGEs) accumulate under chronic hyperglycemia and contribute to type 2 diabetes mellitus (T2DM) complications. Three chemical routes generate AGEs *in vivo*: the Hodge pathway (Amadori rearrangement), the Namiki pathway (Schiff base retroaldol cleavage), and the Wolff pathway (glucose auto-oxidation via Fenton-type chemistry). Each has been extensively characterized, but their integrated behavior in the clinical context — and their relationships to accessible biomarkers — has received less synthetic treatment.

**Objective:**

This paper provides a clinically oriented mechanistic synthesis of the three pathways, identifying their points of convergence and divergence, and proposes — as a hypothesis-generating organizing schema rather than as established biology — a clinical biomarker mapping linking each pathway to routinely accessible laboratory parameters as a basis for prospective validation.

**Approach:**

Narrative mechanistic review synthesizing primary biochemistry, dicarbonyl chemistry, and clinical biomarker literatures.

**Findings:**

The three pathways converge upon a shared reactive dicarbonyl pool comprising methylglyoxal (MGO), glyoxal (GO), and 3-deoxyglucosone (3-DG); upon the GLO1/GLO2 glyoxalase axis (highest catalytic efficiency for MGO; complementary handling of 3-DG by aldose/aldo-keto reductases); and upon RAGE-mediated inflammatory amplification. They diverge in their dominant kinetic drivers (sustained hyperglycemia for Hodge; glycemic variability for Namiki; oxidative-metal milieu for Wolff) and characteristic AGE adducts. Methylglyoxal also arises substantially from glycolytic triose phosphate degradation independent of the AGE synthesis pathways. We propose that routine laboratory parameters — HbA1c × diabetes duration, glycemic variability indices, hs-CRP, ferritin, gamma-glutamyl transferase, and red cell distribution width — may serve as accessible clinical proxies for pathway-specific activity, with the caveat that these mappings require prospective validation.

**Conclusions:**

The Hodge, Namiki, and Wolff pathways are best understood as an integrated AGE synthesis network. This has implications for therapeutic strategy, favoring approaches that act on shared downstream mediators rather than single-pathway interventions, and for the design of pathway-resolved clinical biomarker panels. The proposed mappings and temporal staging are presented as testable hypotheses, not as established clinical algorithms; their validation will require prospective biomarker studies in defined T2DM cohorts.

## 1 Introduction

The Maillard reaction — first described in the context of food browning chemistry — has emerged as one of the most clinically consequential biochemical processes in chronic metabolic disease (1). In the physiological milieu of sustained hyperglycemia, the same chemistry that caramelizes sugars under heat operates slowly but irreversibly across the proteome, lipidome, and nucleome of the diabetic organism, producing a structurally diverse library of adducts, cross-links, and oxidation products collectively designated Advanced Glycation End Products (AGEs) (2, 3). AGE accumulation is now recognized as a central mechanistic contributor to the microvascular and macrovascular complications of type 2 diabetes mellitus (T2DM) (4, 5).

Three principal chemical routes generate AGEs *in vivo*. The Hodge pathway proceeds through Schiff base formation, Amadori rearrangement, and subsequent enolization-fragmentation chemistry to yield reactive dicarbonyl intermediates and stable AGE adducts (1, 6). The Namiki pathway diverges from the Hodge pathway upstream of Amadori rearrangement: the Schiff base undergoes retroaldol cleavage to generate short-chain dicarbonyls directly (7, 8). The Wolff pathway begins outside protein chemistry entirely, with the auto-oxidation of free glucose in solution via Fenton-type metal-catalyzed reactions, producing reactive oxygen species (ROS) and dicarbonyls without requiring protein binding (9, 10).

These three pathways have each been extensively characterized in the biochemistry, food chemistry, and AGE literatures. The shared dicarbonyl chemistry (methylglyoxal, glyoxal, 3-deoxyglucosone) that links them and the shared receptor-mediated downstream amplification (RAGE-NF-κB signaling) have likewise been studied (5, 11, 12). The synthesis we offer here is not the discovery of these convergence points but their integration into a clinically oriented framework: a treatment of the three pathways as elements of a single network with implications for biomarker selection, therapeutic stratification, and the interpretation of routine laboratory findings in T2DM.

HbA1c — glycated hemoglobin — is an important point of orientation. HbA1c is itself an early-stage glycation product (a Schiff base/Amadori-type modification of the β-chain of hemoglobin), most directly reflecting Hodge pathway activity over the prior ~120 days. It is not, mechanistically speaking, an upstream precursor of all three pathways. The Namiki pathway branches from Schiff base intermediates upstream of Amadori formation, and the Wolff pathway proceeds independently of protein binding altogether. HbA1c is therefore a Hodge-dominant biomarker; Namiki and Wolff pathway activities are not directly captured by it, which has long been a source of clinical-mechanistic mismatch when AGE-related complications progress despite acceptable HbA1c values.

This review proceeds as follows. Sections 2–4 examine each pathway mechanistically, with corrected biochemical nomenclature and updated primary references. Section 5 develops the convergence architecture: the shared dicarbonyl pool, the methylglyoxal-dominant glyoxalase clearance bottleneck, and RAGE-mediated amplification. Section 6 introduces the Network-Based Diabetes Care (NBDC) organizing schema as a hypothesis-generating framework for mapping the three pathways to clinically accessible biomarkers, and explicitly distinguishes proposed mappings from validated clinical instruments. Section 7 develops the comparative analysis with existing diagnostic strategies. Section 8 develops the therapeutic implications. Section 9 discusses the limitations of the current synthesis and prioritizes prospective validation work.

## 2 The Hodge pathway

### 2.1 Historical context and nomenclature

The Hodge pathway was formalized by John Hodge in 1953 as a systematic account of the browning reactions occurring when reducing sugars react with amino compounds under heating conditions (1). In biological systems, the relevant temperature is physiological (37 °C) and the timescale extends to months and years of sustained hyperglycemic exposure. Under these conditions, the same chemistry operates as a dominant mechanism of tissue protein modification (2, 3).

The pathway begins with the non-enzymatic condensation of a reducing sugar (glucose, fructose, galactose) with a free amino group. Glycation occurs preferentially at the ϵ-amino group of lysine residues, the α-amino group of protein N-termini, and the guanidino group of arginine residues; nucleic acids (notably the N2 position of guanosine and deoxyguanosine) also serve as glycation substrates. In T2DM, glucose is the predominant sugar substrate by virtue of abundance, although fructose-mediated glycation is increasingly recognized in dietary fructose contexts.

### 2.2 Step-by-step mechanism

#### 2.2.1 Step 1 — Schiff base formation (aldimine)

Glucose reacts with a free amino group (R-NH_2_) to form a hemiaminal intermediate, which spontaneously dehydrates to yield a Schiff base (aldimine, R-N=CH-[CHOH]_4_-CH_2_OH). This is a rapid, reversible reaction with a half-life on the order of hours (6). The Schiff base is the common upstream intermediate shared with the Namiki pathway. HbA1c, measured clinically, reflects the integrated sum of Schiff-base-derived Amadori products on hemoglobin (13).

#### 2.2.2 Step 2 — Amadori rearrangement

The Schiff base undergoes acid-base-catalyzed 1,2-enolization, rearranging to form a more stable ketoamine — the Amadori product (1-amino-1-deoxy-2-ketose) (1, 6). This rearrangement defines the Hodge pathway and distinguishes it from the Namiki pathway, which diverges upstream by competing retroaldol cleavage of the Schiff base. Fructosyl-lysine and HbA1c are clinically familiar Amadori products. It is important to note that Amadori products are early-stage glycation products, not terminal AGEs; this distinction matters when interpreting HbA1c, which is an Amadori product and not an AGE (13, 14).

#### 2.2.3 Step 3 — enolization and dehydration

Amadori products undergo further enolization (1,2- or 2,3-enolization) to generate reactive ene-diol intermediates. These intermediates can dehydrate to form 1,2-ketoaldoses (the IUPAC-recommended nomenclature; the older term “osones” is discouraged), specifically 3-deoxyglucosone (3-DG); they can also undergo retroaldol cleavage to generate shorter-chain dicarbonyls including methylglyoxal (MGO) and glyoxal (GO) (8, 15, 16). This step is a critical convergence junction: the dicarbonyls generated here feed into the same reactive dicarbonyl pool produced by the Namiki and Wolff pathways.

#### 2.2.4 Step 4 — dicarbonyl condensation and AGE formation

The reactive intermediates generated in Step 3 — particularly 3-DG, MGO, and GO — react with additional amino and guanidino groups on proteins to generate stable AGE adducts and cross-links. Important Hodge-pathway-derived AGEs include:

- Pentosidine — a fluorescent lysine-arginine cross-link, measurable in skin collagen and urine (17).
- N-ϵ-Carboxymethyl-lysine (CML) — the most abundant and best-characterized AGE adduct in human tissue, formed predominantly from glyoxal condensation with lysine; a primary RAGE ligand when present as part of a protein adduct (5, 18).
- N-ϵ-Carboxyethyl-lysine (CEL) — formed from MGO condensation with lysine; less abundant than CML in absolute terms but more specific for glycolytic triose-derived MGO and therefore informative as a marker of glycolytic flux-driven dicarbonyl stress (5, 19).
- GOLD and MOLD cross-links — glyoxal-derived (GOLD) and MGO-derived (MOLD) lysine-lysine cross-links, contributing to collagen stiffening and basement membrane thickening characteristic of advanced T2DM (20).

An important biochemical caveat for clinical interpretation: free CML and free CEL have very low affinity for RAGE; effective RAGE binding occurs only when these adducts are presented as structural components of a protein (for example, on serum albumin, collagen, or hemoglobin). Quantification of free vs protein-bound CML and CEL therefore matters for interpreting their pathophysiologic significance.

### 2.3 Kinetics and glycemic dependence

The Hodge pathway is the dominant AGE-synthesis route under conditions of chronic, sustained hyperglycemia. Amadori product accumulation is approximately proportional to time-averaged glucose concentration — the biochemical basis of HbA1c’s clinical correlation with glycemic control (13, 21). A reduction in HbA1c reduces Amadori product formation and proportionally reduces downstream Hodge-pathway AGE generation, which is one mechanistic basis for the long-term complication-reduction benefit of glycemic control documented in landmark intervention trials (22, 23).

### 2.4 Hodge pathway and clinical biomarkers

Beyond HbA1c, biomarkers reflecting integrated Hodge pathway activity over time include the product of HbA1c × diabetes duration as a cumulative-exposure surrogate, urinary pyrraline as a Hodge-derived AGE excretion marker, and skin autofluorescence (SAF) measurements of pentosidine and related fluorescent AGEs. We discuss the relationship of these to other pathway-specific markers in Section 5.

## 3 The Namiki pathway

### 3.1 Historical context and nomenclature

The Namiki pathway was characterized by Masayuki Namiki and Tojiro Hayashi in the 1970s and 1980s through detailed mechanistic studies of Maillard reaction intermediates (7). Where Hodge described the rearrangement of the Schiff base forward to ketoamines, Namiki identified a competing retroaldol cleavage that fragments the Schiff base directly into reactive low-molecular-weight dicarbonyls. The Namiki pathway diverges from the Hodge pathway upstream of Amadori rearrangement, generating dicarbonyls more rapidly than the slower Hodge route (8).

### 3.2 Step-by-step mechanism

#### 3.2.1 Step 1 — Schiff base formation (shared with Hodge)

As in the Hodge pathway, glucose condenses with a free amino group to form a Schiff base aldimine. The Namiki pathway diverges at this point rather than proceeding to Amadori rearrangement.

#### 3.2.2 Step 2 — Schiff base cleavage (the Namiki branch)

Instead of undergoing 1,2-enolization to the Amadori ketoamine, the Schiff base undergoes retroaldol cleavage — a carbon-carbon bond fragmentation that splits the six-carbon sugar chain into shorter-chain aldehydes and dicarbonyls. This cleavage is promoted by:

- Alkaline microenvironments.
- Elevated temperature (relevant in febrile or inflamed tissue).
- Transition metal catalysis (copper, iron — overlapping with Wolff pathway conditions).
- High glucose flux conditions favoring rapid Schiff base turnover.

#### 3.2.3 Step 3 — generation of reactive dicarbonyl intermediates

The cleavage products of the Namiki pathway are primarily glyoxal (GO, a two-carbon 1,2-dicarbonyl) and methylglyoxal (MGO, a three-carbon 1,2-dicarbonyl). Diacetyl (2,3-butanedione, a four-carbon 1,2-diketone) is also produced as a minor product (8, 15). These dicarbonyls are identical to or closely related to those generated downstream in the Hodge pathway and in the Wolff pathway, establishing the shared reactive dicarbonyl pool that is central to the convergence architecture developed in Section 5.

#### 3.2.4 Step 4 — rapid protein adduct formation and AGE generation

Dicarbonyls generated by Namiki cleavage react with amino and guanidino groups on proteins with kinetics far faster than the intact Schiff base or Amadori product reactions (5). MGO reacts preferentially with the guanidino group of arginine residues to form MG-H1 (methylglyoxal-derived hydroimidazolone-1), the most abundant MGO-derived AGE in human tissue (11, 24). GO reacts with lysine to form CML and with arginine to form glyoxal-derived hydroimidazolone (G-H1). As with CML and CEL, free MG-H1 and G-H1 have low intrinsic RAGE affinity; their pathophysiologic significance arises predominantly when present as protein adducts (5).

### 3.3 Kinetics and glycemic-variability dependence

The Namiki pathway is particularly active under conditions of acute hyperglycemia and oxidative stress — states that favor rapid Schiff base turnover and metal-catalyzed fragmentation (8, 25). It is therefore not strictly proportional to HbA1c. Patients with high glycemic variability — large postprandial glucose excursions with relative fasting normoglycemia — may have disproportionate Namiki pathway activity relative to their HbA1c, providing one mechanistic explanation for the clinical observation that complications can progress despite acceptable HbA1c when glycemic variability is elevated (26, 27).

### 3.4 Namiki pathway and clinical biomarkers

Plasma MGO (measured by LC-MS/MS or stable-isotope dilution assays) and urinary MG-H1 are the most direct biomarkers of Namiki pathway activity. Continuous glucose monitoring-derived metrics — coefficient of variation, mean amplitude of glycemic excursions (MAGE), time-in-range — provide indirect kinetic indicators of Namiki pathway substrate availability. We discuss accessible routine-laboratory candidates for Namiki pathway monitoring in Section 6.

## 4 The Wolff pathway

### 4.1 Historical context and nomenclature

The Wolff pathway — named for Simon Wolff, who characterized glucose auto-oxidation mechanisms in the late 1980s — represents a fundamentally different entry point into AGE synthesis (9, 10). Where the Hodge and Namiki pathways both begin with protein-glucose condensation, the Wolff pathway begins with the direct auto-oxidation of free glucose in solution, independent of protein binding. This makes it the only AGE-synthesis route that can proceed in extracellular fluid, plasma, and intracellular aqueous compartments without requiring a protein scaffold.

### 4.2 Step-by-step mechanism

#### 4.2.1 Step 1 — glucose enolization and transition metal catalysis

In aqueous solution, glucose exists in equilibrium between its ring (pyranose/furanose) and open-chain (acyclic) forms. The open-chain form undergoes enolization to produce a 1,2-enediol intermediate. This enolization is catalyzed by transition metal ions — particularly Cu^2+^ and Fe^3+^ — which accept electrons from the enediol, reducing to Cu^+^ and Fe^2+^ while generating a glucose radical (9).

#### 4.2.2 Step 2 — reactive oxygen species generation

The reduced metal ions (Cu^+^, Fe^2+^) react with molecular oxygen in Fenton-type reactions to generate superoxide radical (O_2_•^-^) and hydrogen peroxide (H_2_O_2_). This creates a localized pro-oxidant microenvironment, constituting a direct link between hyperglycemia and oxidative stress that operates independently of polyol pathway flux, electron transport chain leakage, or mitochondrial uncoupling (10, 28).

#### 4.2.3 Step 3 — reactive dicarbonyl generation

The oxidized glucose intermediates undergo further fragmentation to generate reactive dicarbonyls — principally glyoxal (GO), methylglyoxal (MGO), and glucosone (a ketoaldehyde) (9, 15). As in the Namiki pathway, these products converge upon the same reactive dicarbonyl pool generated by the Hodge pathway, demonstrating biochemical integration of all three routes.

#### 4.2.4 Step 4 — AGE formation and oxidative AGE variants

The dicarbonyls and ROS generated by the Wolff pathway react with proteins to form AGEs — primarily CML (from glyoxal + lysine) and MG-H1 (from MGO + arginine). The simultaneous generation of ROS creates a class of oxidation-dependent AGEs sometimes designated glycoxidation products: AGEs requiring both glycation and oxidative conditions for their formation. CML and pentosidine are the best-characterized glycoxidation products and are therefore preferential markers of integrated Wolff pathway and Hodge oxidative activity, though CML is also produced by MGO-independent oxidation of polyunsaturated fatty acids and is therefore not strictly pathway-specific (5, 29).

### 4.3 Kinetics and oxidative-milieu dependence

The Wolff pathway has a non-linear relationship to ambient glucose concentration. Because it depends on transition metal availability and oxygen tension in addition to glucose concentration, it is disproportionately active in states of oxidative stress, inflammation, and metal dyshomeostasis — conditions that themselves develop progressively in advancing T2DM (10, 30). This generates a positive feedback loop: Wolff pathway activity generates ROS; ROS promote oxidative stress; oxidative stress increases free transition metal availability (via ferritin degradation and copper release from ceruloplasmin); free metals further accelerate Wolff pathway kinetics (31, 32).

### 4.4 Wolff pathway and clinical biomarkers

Direct measurement of Wolff pathway activity in clinical practice is limited. Surrogate markers reflecting the oxidative-metal milieu in which Wolff chemistry is amplified include systemic inflammation markers (hs-CRP), iron-metabolism markers (ferritin, transferrin saturation), and oxidative DNA damage markers (8-OHdG; specialty laboratory). The proposed mapping of these markers to Wolff pathway activity is discussed in Section 6 as a hypothesis-generating schema.

## 5 Convergence architecture: where the three pathways meet

### 5.1 The shared reactive dicarbonyl pool

All three AGE synthesis pathways converge upon a common pool of reactive 1,2-dicarbonyl species comprising methylglyoxal (MGO), glyoxal (GO), and 3-deoxyglucosone (3-DG) (**Table 1**) (5, 11, 15). Importantly, MGO is also generated continuously through a fourth, AGE-independent route: the spontaneous degradation of glycolytic triose phosphate intermediates (dihydroxyacetone phosphate, DHAP, and glyceraldehyde-3-phosphate, GA3P) (33, 34). Under high glycolytic flux conditions or impaired glyceraldehyde-3-phosphate dehydrogenase function, this metabolic-flux source contributes substantially to total cellular MGO, particularly in tissues with high glycolytic activity such as endothelium and erythrocytes (33).

**Table 1:** Reactive dicarbonyl intermediates generated by the three AGE synthesis pathways.

Dicarbonyl	Hodge pathway	Namiki pathway	Wolff pathway
Methylglyoxal (MGO)	From Amadori product retroaldol cleavage; from 1,2-enediol of 3-DG	Primary product of Schiff base cleavage and fragmentation	From glucose auto-oxidation; from enediol fragmentation
Glyoxal (GO)	From Amadori product oxidative degradation via 2,3-enolization	Co-product of Schiff base retroaldol cleavage	Primary auto-oxidation product; Fenton-derived
3-Deoxyglucosone (3-DG)	Major product of Amadori 1,2-enolization–dehydration	Minor product; from Schiff base fragments	Secondary product; from glucosone reduction

This convergence has therapeutic implications: agents that act on the dicarbonyl pool itself — direct dicarbonyl scavengers such as pyridoxamine, carnosine, and certain bioflavonoids — should in principle intercept the output of all three pathways simultaneously. We return to this point in Section 8.

### 5.2 The glyoxalase system as the principal MGO clearance route

The principal enzymatic defense against intracellular MGO accumulation is the glyoxalase system, comprising glyoxalase I (GLO1; lactoylglutathione lyase) and glyoxalase II (GLO2; hydroxyacylglutathione hydrolase), which together convert MGO to D-lactate in a glutathione (GSH)-dependent two-step reaction (11, 35). The catalytic efficiency of GLO1/GLO2 is greatest for MGO; clearance of GO occurs at substantially lower efficiency, and 3-DG is handled predominantly not by the glyoxalase system but by aldose reductase and aldo-keto reductases of the AKR family (35, 36). The glyoxalase system is therefore best characterized as the principal clearance route for MGO specifically, with complementary handling of other dicarbonyls by parallel reductase systems — rather than as an equivalent clearance system for all reactive dicarbonyl species.

Clinical relevance of glyoxalase function. Polymorphisms in GLO1 (notably the A111E variant) have been associated with altered enzyme activity and modified microvascular complication risk in T2DM cohorts. GLO1 expression is upregulated by Nrf2-dependent transcriptional programs and modulated by AMPK signaling, providing molecular pathways through which agents discussed in Section 8 (notably metformin) may exert pleiotropic anti-AGE effects.

### 5.3 Shared receptor target — RAGE

All three pathways generate AGE adducts that serve, when protein-bound, as ligands for the Receptor for Advanced Glycation End Products (RAGE) (12, 37). RAGE is a multi-ligand pattern-recognition receptor that responds to AGE-modified proteins largely independently of the pathway of origin of the modifying dicarbonyl. RAGE activation initiates intracellular signaling through MAPK and NF-κB pathways, driving transcription of pro-inflammatory genes (TNF-α, IL-6, VCAM-1, ICAM-1, tissue factor) and amplifying ROS generation through NADPH oxidase activation (37, 38). RAGE-mediated amplification is therefore pathway-agnostic: it integrates AGE burden from Hodge, Namiki, and Wolff pathway origins into a single inflammatory readout.

### 5.4 Differential AGE signatures across pathways

Despite convergent dicarbonyl chemistry, the three pathways generate distinguishable AGE signatures that — with appropriate caveats about overlap and protein-binding state — can in principle support pathway-resolved monitoring.

These signatures overlap substantially, particularly for CML and pentosidine, which are produced by both Hodge oxidative degradation and Wolff pathway chemistry. CEL, in contrast, has greater specificity for triose-derived (glycolytic flux) MGO. Pathway-resolved interpretation therefore requires biomarker panels rather than single markers; we develop this point in Section 6. The pathway-characteristic AGE adducts and their accessible biomarkers are summarized in **Table 2**.

**Table 2:** Pathway-characteristic AGE adducts and their accessible biomarkers.

Pathway	Dominant intermediate	Signature AGE adduct(s)	Substrate	Accessible biomarker
Hodge	3-Deoxyglucosone (3-DG)	Pentosidine; pyrraline; CML (also Wolff)	Lysine; collagen	Skin autofluorescence; urinary pyrraline; HbA1c × duration
Namiki	Methylglyoxal (MGO)	MG-H1 (hydroimidazolone); CEL (triose-derived)	Arginine; lysine	Plasma MGO; urinary MG-H1; CGM-derived variability indices
Wolff	Glyoxal (GO) + ROS	CML; G-H1; pentosidine; oxidative-AGE adducts	Lysine; arginine; lipids	Serum CML; 8-OHdG; F2-isoprostanes

### 5.5 Temporal sequencing of pathway activity — a proposed hypothesis

The three pathways are unlikely to be equally active across all stages of T2DM. We propose, as a hypothesis requiring prospective validation rather than as established biology, that pathway dominance shifts across the trajectory of metabolic disease:

- Pre-diabetes/early T2DM: Wolff pathway activity may be disproportionately prominent, driven by oxidative-metal milieu (elevated ferritin, hs-CRP) often present in insulin-resistant states even before HbA1c crosses diagnostic thresholds. This prediction is consistent with epidemiological observations linking inflammation and iron-metabolism markers to incident T2DM, but the proposed Wolff-pathway-dominance attribution is itself a hypothesis.
- Established T2DM (HbA1c ≈ 7–9%): Hodge pathway activity becomes quantitatively dominant due to sustained Amadori product accumulation. This is the disease window in which HbA1c-lowering most directly affects AGE generation.
- Advanced T2DM with complications: Namiki pathway activity may rise with increasing glycemic variability (particularly in insulin-treated patients with hypoglycemia-hyperglycemia cycling), while Wolff pathway activity is amplified by the pro-oxidant tissue environment of established microvascular disease.

This proposed temporal staging is a hypothesis-generating organizing schema. Prospective longitudinal biomarker studies — particularly studies pairing CGM-derived variability metrics with pathway-specific biomarkers (plasma MGO, urinary MG-H1, ferritin trajectories) — are required to test it. Until such validation is available, the staging should not be treated as an established clinical algorithm.

## 6 An organizing schema: pathway-resolved clinical biomarkers

### 6.1 Purpose and explicit caveats

The primary therapeutic implication of the three-pathway integration developed in Sections 2–5 is that no single biomarker — including HbA1c — can capture total AGE synthesis activity. The Hodge pathway is best reflected in HbA1c-derived metrics; the Namiki pathway is best reflected in glycemic variability and direct dicarbonyl measurements; the Wolff pathway is best reflected in inflammation and iron-metabolism markers. A pathway-resolved interpretation therefore requires combining clinically accessible biomarkers into a panel.

We present below an organizing schema for such a panel, drawn from biomarkers routinely available on standard laboratory panels (CBC, CMP) and from continuous glucose monitoring data. We emphasize at the outset:

- This schema is hypothesis-generating, not validated. The proposed mappings between specific biomarkers and specific pathways have a mechanistic rationale developed below, but they have not been prospectively validated in clinical cohorts. They are presented as testable hypotheses, not as a clinical scoring instrument.
- Several proposed mappings have alternative explanations. Elevated GGT, for example, has multiple potential interpretations beyond the glyoxalase-system-load hypothesis we develop here. The schema should therefore not be applied clinically without acknowledgement of these alternatives.
- Validation will require prospective biomarker studies. We outline the design parameters of such studies in Section 9.

### 6.2 Proposed pathway-biomarker mappings

Within these caveats, the following mappings have a defensible mechanistic basis from the convergence chemistry developed in Section 5 (**Table 3**):

**Table 3:** Proposed clinical biomarker mappings to AGE synthesis pathways (hypothesis-generating).

Pathway	Proposed biomarker	Mechanistic rationale	Caveats/alternatives
Hodge (sustained)	HbA1c × diabetes duration; SAF; urinary pyrraline	HbA1c reflects Amadori product on hemoglobin; duration captures cumulative exposure; SAF reflects pentosidine in skin collagen	HbA1c reflects only Hodge activity, not Namiki/Wolff; SAF assays are not yet standardized; urinary pyrraline limited by interlaboratory variability
Namiki (variability-driven)	CGM-derived variability indices (CV, MAGE, time-in-range); plasma MGO; urinary MG-H1; RDW (proposed mechanism in companion review)	Postprandial excursions drive Schiff base turnover and retroaldol cleavage; MGO modifies erythrocyte membrane proteins, contributing to anisocytosis	Plasma MGO and MG-H1 require specialized assays; RDW elevation has multiple causes (nutritional, inflammatory) that must be excluded
Wolff (oxidative-metal)	hs-CRP; ferritin; transferrin saturation; uric acid; GGT (proposed mechanism in companion review)	Oxidative-metal milieu amplifies Fenton-type chemistry; GGT reflects hepatic GSH turnover under glyoxalase-system load	hs-CRP and ferritin are non-specific (general inflammation, iron storage); GGT has alternative interpretations (hepatic, alcohol, medication); none of these is pathway-specific in isolation
Pathway-integrated	Serum CML; CEL (when available)	Integrated AGE burden output; CEL more specific to triose-derived MGO	Free vs protein-bound CML/CEL must be distinguished for RAGE-relevant interpretation

The biomarker mappings for GGT and RDW deserve particular comment. We have developed the mechanistic rationale for these proposed mappings — GGT as a marker of glyoxalase-system glutathione turnover and RDW as a marker of erythrocyte membrane MGO modification — in companion mechanistic reviews currently in preparation for separate publication. Both proposed mappings require prospective validation; until such validation is available, GGT and RDW should be interpreted with their established clinical meanings rather than as pathway-specific AGE markers.

### 6.3 Distinction from a validated scoring instrument

The schema presented above is not a scoring algorithm. It is an organizing framework for considering routine biomarkers in the context of the three-pathway AGE chemistry. We deliberately do not present weighted composite scores, threshold values, or therapeutic-decision algorithms in this paper. The development of any such instrument would require independent prospective validation in adequately powered cohorts, which is outside the scope of the present mechanistic synthesis.

## 7 Comparison with existing diagnostic strategies

The schema developed in Section 6 should be situated relative to existing clinical diagnostic strategies for diabetes and metabolic risk. We do not claim it replaces these strategies; rather, the question is what additional information it might contribute.

### 7.1 HbA1c

HbA1c remains the cornerstone of glycemic monitoring in T2DM, with extensive validation as a predictor of microvascular complications (22, 23). As discussed in Section 2, HbA1c reflects Hodge-pathway-dominant glycation activity over approximately 120 days. Its limitations — including insensitivity to glycemic variability, dependence on erythrocyte lifespan, and racial/ethnic variation in HbA1c-glucose relationships — are well documented (39, 40). The Namiki and Wolff pathway activities described in this review are not directly captured by HbA1c, providing one mechanistic basis for the well-known phenomenon of complication progression despite acceptable HbA1c values.

### 7.2 Insulin resistance indices

HOMA-IR (Homeostasis Model Assessment of Insulin Resistance), the Matsuda Index, and the triglyceride-to-glucose ratio are widely used insulin-resistance indices (41, 42). These indices quantify the systemic milieu in which the Wolff pathway operates — insulin-resistant, pro-inflammatory, and frequently iron-replete states are conducive to oxidative chemistry — but they do not directly measure AGE synthesis or dicarbonyl burden. They are complementary to, not redundant with, the pathway-specific biomarkers proposed in Section 6.

### 7.3 Direct AGE measurements

Direct measurement of AGE adducts — serum CML, plasma MGO, urinary MG-H1, skin autofluorescence — is feasible but currently limited to research laboratories or specialty clinical centers in most healthcare systems (43, 44). The advantage of the schema in Section 6 is that it draws on biomarkers already universally available on routine panels (HbA1c, hs-CRP, ferritin, GGT, RDW); the disadvantage is that none of these proxies measures AGE chemistry directly.

### 7.4 GLO1 activity assays

Direct measurement of GLO1 activity in erythrocyte lysates is technically feasible but is not part of standard clinical practice and is not available on routine panels in any large-scale healthcare system. Its role at present is in research settings, where it may serve as a target-engagement biomarker for glyoxalase-system-modulating interventions.

## 8 Therapeutic implications

### 8.1 Strategy 1 — sustained Hodge pathway suppression via glycemic control

Sustained reduction of mean glucose exposure — through any pharmacological or lifestyle mechanism — reduces Amadori product accumulation and proportionally reduces downstream Hodge-pathway AGE generation (22, 23). This is the mechanism by which long-term HbA1c control reduces microvascular complication risk in randomized trials, and it remains the primary therapeutic intervention against AGE-mediated tissue injury.

### 8.2 Strategy 2 — glycemic variability reduction (Namiki pathway)

Interventions that reduce postprandial glycemic excursions — including time-in-range optimization, low-glycemic-index dietary patterns, and GLP-1 receptor agonists that slow gastric emptying — should reduce Namiki pathway activity by limiting the substrate availability for Schiff base retroaldol cleavage (27, 45). The clinical evidence base for variability-reduction-specific outcomes (independent of HbA1c lowering) is still developing.

### 8.3 Strategy 3 — Wolff pathway suppression

Reducing the oxidative-metal milieu in which Wolff pathway chemistry is amplified is mechanistically plausible but has limited direct clinical-trial support (30, 32). Iron-metabolism moderation (avoiding excess heme iron intake; treating iron overload), polyphenol intake (quercetin, hesperidin, epigallocatechin gallate; partial transition-metal chelators with documented anti-AGE activity *in vitro*), and antioxidant strategies have a mechanistic rationale but await definitive clinical efficacy data (46, 47).

### 8.4 Strategy 4 — glyoxalase system support and direct dicarbonyl trapping

The convergence architecture developed in Section 5 implies that interventions acting on shared downstream mediators — the dicarbonyl pool itself or the glyoxalase clearance system — should reduce AGE generation across all three pathways. This is a mechanistic argument, not yet a comparative-efficacy demonstration; comparative trials with pathway-specific interventions have generally not been performed.

- Metformin — the most widely used T2DM agent, with documented effects on GLO1 expression and MGO levels through AMPK-mediated mechanisms and effects on Nrf2 signaling, additive to its primary glucose-lowering action (48, 49).
- N-acetylcysteine (NAC) — GSH precursor, replenishing the GLO1 cofactor pool; reduces plasma MGO in experimental models (50).
- Alpha-lipoic acid — regenerates GSH from GSSG and has direct antioxidant activity; modest evidence for symptomatic improvement in diabetic peripheral neuropathy (51).
- Pyridoxamine — direct trapping of MGO and GO; reduces urinary MG-H1 in clinical trials of early diabetic nephropathy (52, 53).
- Carnosine and beta-alanine — carnosine reacts with MGO at its histidine residue; beta-alanine increases tissue carnosine; modest reductions in plasma MGO in human studies (54).
- Bioflavonoids (quercetin, hesperidin, epigallocatechin gallate) — reduce plasma MGO and improve markers of glycation in some randomized trials; mechanism includes both direct dicarbonyl trapping and polyphenol-mediated metal chelation (46, 47).

### 8.5 A note on AGE-targeting agents and their translational history

Several agents historically discussed as AGE-targeted interventions have had complex translational trajectories that should be acknowledged in any clinical discussion.

- Aminoguanidine (pimagedine) — a hydrazine compound that reacts with reactive dicarbonyls to prevent AGE cross-link formation; demonstrated efficacy in animal models of diabetic nephropathy and retinopathy. Clinical development was discontinued due to adverse effects (anti-glomerular basement membrane antibody formation, vasculitis) in the ACTION-1 trial. Aminoguanidine is therefore not in current clinical use; it remains a research tool only (55).
- Alagebrium (ALT-711) — an AGE cross-link breaker designed to cleave established collagen cross-links; phase II trials demonstrated improvements in arterial compliance, but development was discontinued for commercial reasons (56).
- RAGE antagonists — azeliragon (TTP488) and related compounds entered clinical trials for Alzheimer’s disease and related indications; results have been mixed and clinical use remains investigational (57).

The history of AGE-targeted drug development should temper claims that any particular dicarbonyl-targeting strategy is established as superior to existing therapies. The convergence-based mechanistic rationale developed in this review is sound; demonstrating clinical superiority of dicarbonyl-pool interventions over individual-pathway interventions requires direct comparative trials that have not been done.

## 9 Limitations and prospective validation

The synthesis presented in this review has several limitations that should be made explicit.

### 9.1 Mechanistic claims rest predominantly on *in vitro* and animal-model evidence

The biochemistry of the Hodge, Namiki, and Wolff pathways has been characterized predominantly in chemical kinetics studies, cell culture systems, and animal models. The relative quantitative contribution of each pathway *in vivo* in human T2DM, at specific stages of disease, has not been definitively established. Prospective biomarker studies pairing pathway-specific markers (plasma MGO, urinary MG-H1, ferritin trajectories, CGM-derived variability) with clinical outcomes are required.

### 9.2 The biomarker mappings in section 6 require prospective validation

The proposed mappings between routine biomarkers and AGE pathway activity have a defensible mechanistic rationale but have not been prospectively validated. Studies linking GGT, RDW, hs-CRP, and ferritin trajectories to direct measurements of MGO and AGE adducts in defined T2DM cohorts are needed before these mappings can be considered clinically actionable. We make no claim that the schema in Section 6 constitutes a clinical scoring instrument.

### 9.3 The proposed temporal pathway hierarchy is a hypothesis

The Wolff → Hodge → Namiki dominance shift across T2DM stages is presented as a hypothesis-generating organizing schema, not as established biology. Some elements have epidemiological support (e.g., elevated inflammatory and iron-metabolism markers preceding HbA1c diagnostic thresholds); others, particularly the late-stage Namiki dominance attribution, require explicit prospective testing.

### 9.4 Comparative therapeutic efficacy claims are limited by absence of direct trials

The argument that dicarbonyl-pool interventions should affect AGE generation across all three pathways is mechanistically sound but does not constitute evidence of clinical superiority over individual-pathway interventions. The convergence architecture provides a rationale for comparative studies; it does not substitute for them.

### 9.5 Priorities for future research

The validation work most likely to convert the synthesis presented here from hypothesis to established clinical framework includes:

- Pathway-specific biomarker cohorts: prospective measurement of plasma MGO, urinary MG-H1, GGT, RDW, ferritin, and CGM-derived variability in defined T2DM populations stratified by disease duration, with longitudinal correlation to clinical outcomes.
- Pharmacodynamic studies: evaluation of biomarker responses to interventions with established or hypothesized glyoxalase-targeting effects (metformin, NAC, pyridoxamine, beta-alanine), to establish accessible target-engagement readouts.
- Comparative therapeutic trials: head-to-head evaluation of single-pathway versus dicarbonyl-pool interventions, with hard clinical endpoints.
- Standardization of AGE assays: interlaboratory standardization of plasma MGO, urinary MG-H1, and skin autofluorescence measurements to support multicenter validation.

## 10 Conclusions

The Hodge, Namiki, and Wolff pathways of AGE synthesis are best understood as elements of an integrated chemical network rather than as parallel independent routes. They share a reactive dicarbonyl pool comprising methylglyoxal, glyoxal, and 3-deoxyglucosone; they share — for MGO at least — a single principal enzymatic clearance system, the GLO1/GLO2 glyoxalase axis; and they converge on RAGE-mediated inflammatory amplification of their downstream consequences. Methylglyoxal is also generated by glycolytic triose phosphate degradation independent of the AGE synthesis pathways, broadening the chemistry of the dicarbonyl pool beyond the three classical AGE routes.

These convergence points have implications for clinical biomarker selection (favoring pathway-resolved panels over HbA1c monotherapy), for therapeutic strategy (favoring interventions on shared downstream mediators), and for the interpretation of routine laboratory findings in T2DM. We have proposed an organizing schema mapping accessible biomarkers to pathway activities, with the explicit caveat that the proposed mappings are hypothesis-generating and require prospective validation.

The synthesis offered here is not the discovery of these convergence points — they have been individually documented in the AGE literature for decades. It is the integration of these mechanistic elements into a clinically oriented framework that addresses a question of practical importance: what should the practicing clinician make of the routine biomarkers available at every patient visit, in light of what is known about AGE chemistry? The framework we propose is offered as a starting point for that question, not as a settled answer.

## Data Availability

The original contributions presented in the study are included in the article/supplementary material. Further inquiries can be directed to the corresponding author.
